# Editorial: Genomic Assessment of Adaptive Potential of Populations to Environmental Challenges: Applications to Management and Conservation

**DOI:** 10.1111/eva.70364

**Published:** 2026-11-10

**Authors:** Maren Wellenreuther, David Chagné, Juntao Hu, Sam Yeaman, Yiyong Chen, Lesley T. Lancaster, Santiago C. González‐Martínez

**Affiliations:** ^1^ New Zealand Institute for Bioeconomy Science Lincoln New Zealand; ^2^ School of Biological Sciences The University of Auckland Auckland New Zealand; ^3^ Department of Ecology and Evolutionary Biology, School of Life Sciences Fudan University Shanghai China; ^4^ Department of Biological Sciences University of Calgary Calgary Alberta Canada; ^5^ Research Center for Eco‐Environmental Sciences Chinese Academy of Sciences Beijing China; ^6^ School of Biological Sciences The University of Aberdeen Aberdeen UK; ^7^ BIOGECO, INRAE, University Bordeaux Cestas France

## Abstract

Rapid environmental change poses unprecedented challenges for species persistence, threatening not only population size but the genomic adaptive variation required for evolutionary responses. Understanding this adaptive potential is crucial for effective management and conservation strategies, as it determines a population's capacity to cope with current and future climate shifts, habitat loss, emerging diseases and other environmental pressures. This Special Issue is therefore timely in the context of advancing genetic diversity monitoring recommendations (e.g., UN Convention on Biological Diversity Target 4), bringing together 40 original papers, perspectives and reviews that link genomic insights to conservation status and practice. The contributions span empirical studies, mechanistic investigations, theoretical frameworks and applied case studies. Together, these studies highlight how genomic approaches can illuminate the mechanisms and distribution of adaptive variation and inform strategies to maintain population resilience and guide effective management under environmental change.

## 1 Introduction

Rapid, human‐induced environmental change is creating unprecedented challenges for conservation biology, evolutionary research and resource management (Umina et al. 2005; Hoegh‐Guldberg et al. 2007; Montes‐Hugo et al. 2009). Climate change, habitat loss and fragmentation, overexploitation, biological invasions and environmental pollution are driving population declines and extinctions, phenotypic change, and biodiversity loss worldwide (Urban et al. 2016). In some cases, entire species have disappeared within only a few decades, underscoring that biodiversity collapse is no longer a future concern but an ongoing global reality (Parmesan and Yohe 2003; Perry et al. 2005; Chen et al. 2011; Humphreys et al. 2019). An important aspect of how these pressures increase extinction risk is their role in eroding adaptive genomic diversity, increasing inbreeding and genetic load, and disrupting genetic connectivity, thereby reducing both demographic stability and the evolutionary capacity of populations to respond to rapid environmental change (Aguirre‐Liguori et al. 2021). Many of these drivers are projected to intensify and interact synergistically over coming decades, further accelerating rates of biodiversity loss. Against this backdrop, the Special Issue *Genomic Assessment of Adaptive Potential of Populations to Environmental Challenges: Applications to Management and Conservation* is both timely and urgent.

This Special Issue brings together empirical, theoretical, and methodological studies examining how genomic variation shapes (and is shaped by) population responses to environmental stressors, and how these insights can be translated into conservation and management practice. Collectively, the contributions provide mechanistic insights into adaptation, evaluate emerging genomic tools and novel methodologies, and explore practical pathways for maintaining population resilience under environmental change. Several studies also critically assess the uncertainties and implementation challenges associated with applying genomic approaches in real‐world conservation and management contexts.

The papers span five interconnected themes: (1) local adaptation and climate vulnerability; (2) genomic offsets and predictive approaches for forecasting maladaptation; (3) mechanisms of adaptation and functional genomics; (4) population genomics, connectivity and demographic history; and (5) applied conservation and restoration, including assisted gene flow, population monitoring and adaptive management frameworks. Across these themes, the Special Issue highlights the growing potential of genomics to improve predictions of population persistence, guide conservation interventions, and support evidence‐based management under uncertainty.

The timing of this collection is particularly critical. As environmental change accelerates, understanding both within‐species evolutionary potential and among‐species phylogenetic diversity is becoming essential for predicting how populations, communities and ecosystems will respond to global change, and for informing decisions related to sustainable production, ecosystem health and human well‐being (De Meester et al. 2024). Conserving adaptive potential is increasingly important for maintaining biodiversity, ecosystem function, and long‐term population persistence. This urgency is reflected in the rapidly accumulating number of studies that investigate the genomic basis of adaptation and climate resilience in natural populations (Figure 1). By integrating research across taxa, ecosystems and analytical frameworks (Figure 2), this Special Issue provides a timely overview of how evolutionary approaches can inform conservation and management in the Anthropocene and support strategies aimed at preserving evolutionary resilience in a rapidly changing world.

**FIGURE 1:**
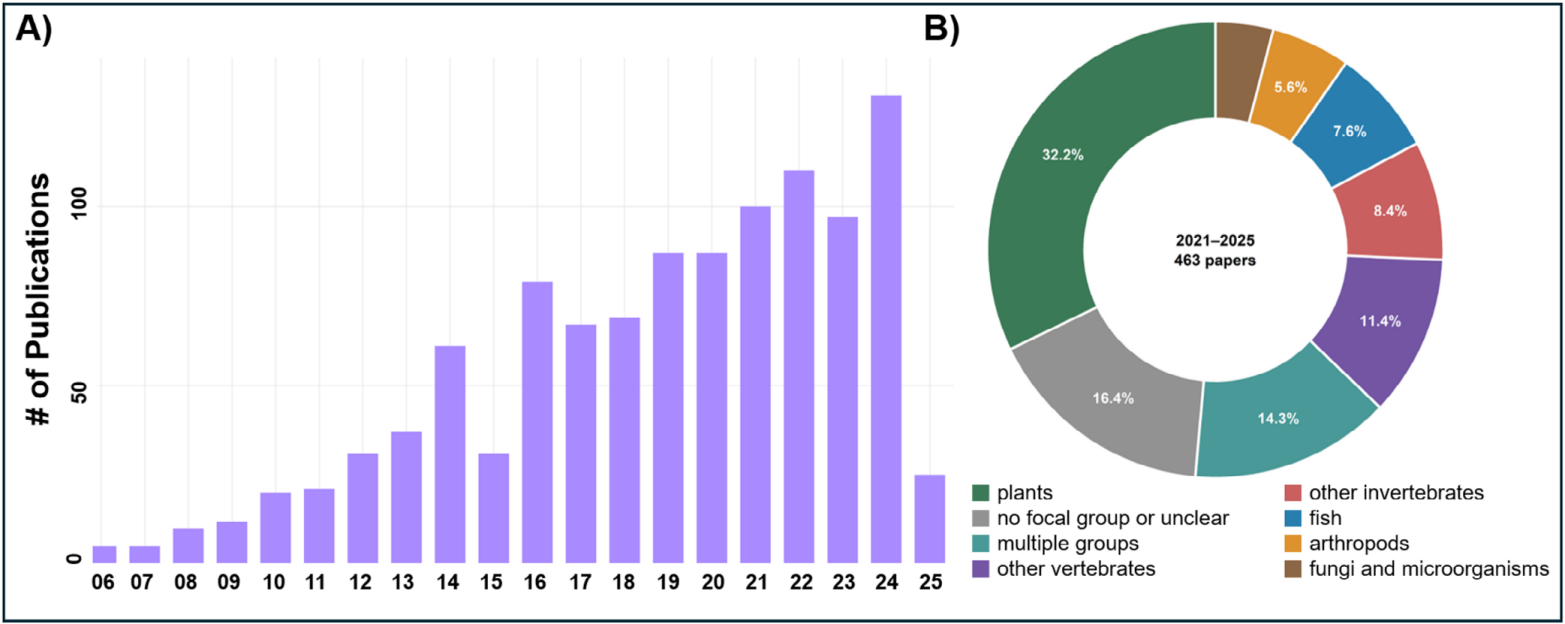
Web of Science publication trends and focal organism groups. We searched the Web of Science Core Collection for publications containing the topic terms ‘adaptive potential’, ‘evolutionary potential’, or ‘genomic vulnerability’. Results were restricted to English‐language research articles and letters. Reviews, book chapters, proceedings papers, meeting abstracts and records from unrelated subject categories and research areas were excluded. After filtering and removal of one duplicate, 1092 unique publications were retained. (A) Annual publication output from 2006 to 2025 (shown as 06–25; *n* = 1085). (B) Broad focal organism groups represented in publications from 2021 to 2025 (*n* = 463): 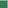 plants; 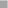 no focal group or unclear; 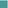 multiple groups; 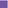 other vertebrates; 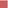 other invertebrates; 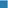 fish; 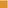 arthropods; 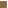 fungi and microorganisms. Percentages indicate the relative contribution of each group. Organism groups were classified from publication titles, abstracts and keywords.

**FIGURE 2:**
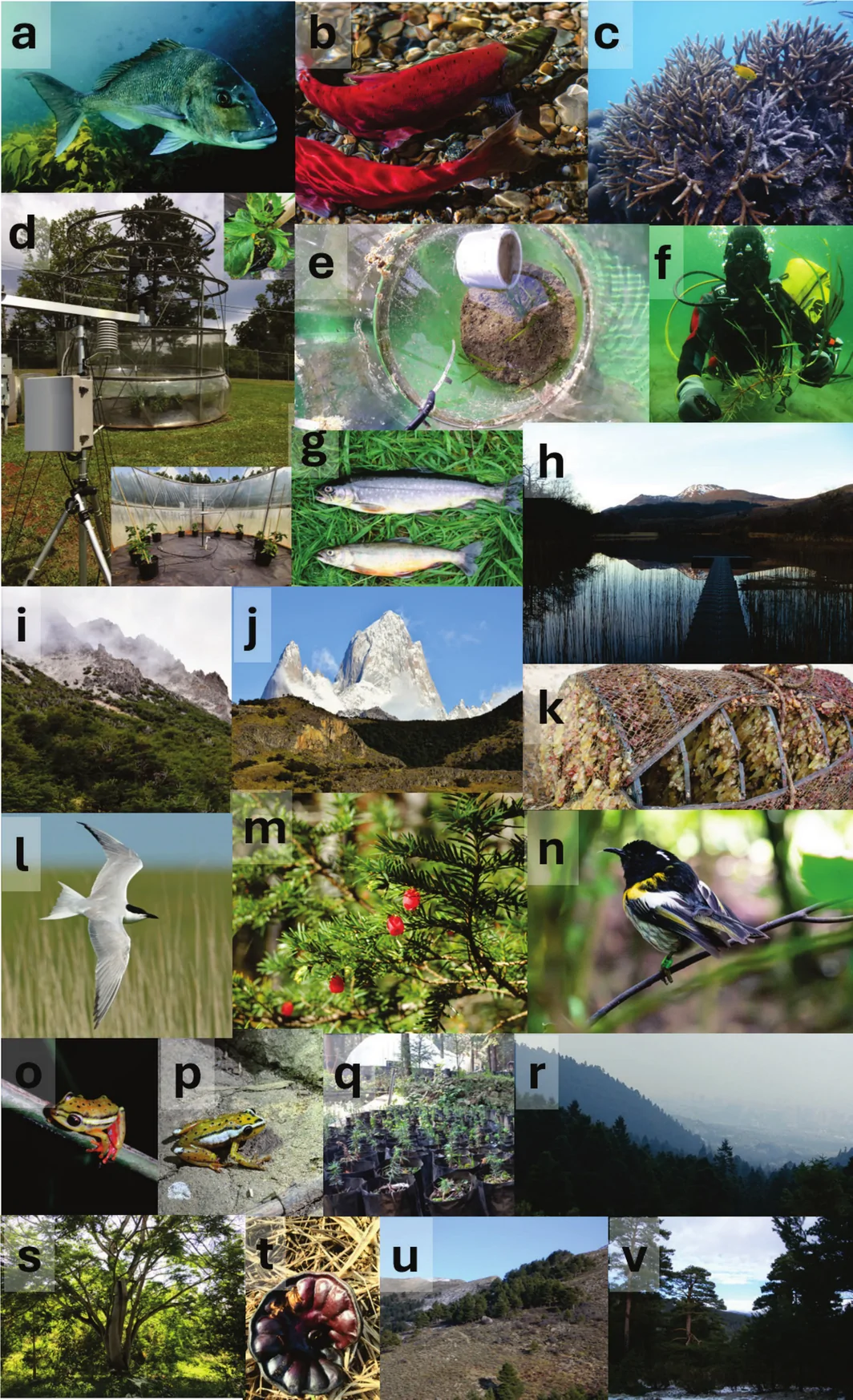
Collage illustrating the diversity of study systems featured in this Special Issue, spanning a wide range of taxa and ecosystems, including forests, mountain landscapes, rivers, lakes, coral reefs and coastal environments. Together, these studies highlight the taxonomic, ecological, and geographic breadth of evolutionary genomic research aimed at understanding adaptation, resilience and responses to environmental change in natural and managed populations worldwide. Panels show: (a) snapper, *Chrysophrys auratus* (photo credit: Ben Harris); (b) spawning kokanee salmon, *Oncorhynchus nerka* (photo credit: Craig Goodwin); (c) *Acropora muricata* coral from Mauritius (photo credit: Oliver Selmoni); (d) the *Xanthomonas*–pepper pathosystem (photo credit: Ivory Russell); (e) eelgrass, *Zostera marina* , in a mesocosm experiment (photo credit: Lars Jahnke); (f) a diver collecting *Z. marina* for mesocosm experiments (photo credit: Christian Pansch‐Hattich); (g) Arctic charr, *Salvelinus alpinus* , from Loch Seil, Scotland (photo credit: Sam Fenton); (h) freshwater habitat associated with Arctic charr research (photo credit: Kathryn Elmer); (i, j) *Nothofagus pumilio* forests and their associated mountain landscapes (photo credit: Jill Sekely); (k) a dense aggregation of the invasive ascidian *Ciona robusta* attached to aquaculture substrate (photo credit: Yiyong Chen); (l) gull‐billed terns, *Gelochelidon nilotica* (photo credit: Anna Schnelle); (m) red arils of English yew, *Taxus baccata* (photo credit: Maria Mayol); (n) hihi/stitchbird, *Notiomystis cincta* (photo credit: Hui Zhen Tan); (o, p) *Hyperolius mitchelli* (photo credits: Chris Barratt and Kat Stewart, respectively); (q) experimental seedlings of sacred fir, *Abies religiosa* (photo credit: Sebastian Arenas); (r) sacred fir forest, a conifer ecosystem endemic to the mountains of central Mexico (photo credit: Sebastian Arenas); (s) an *Enterolobium cyclocarpum* tree and (t) its fruit (photo credit: Francisco Velásquez Puentes); and (u, v) Scots pine, *Pinus sylvestris* , in its natural habitat (photo credit: Santiago C. González‐Martínez).

## 2 Advances From Contributions to This Special Issue

### 2.1 Local Adaptation and Climate Vulnerability

Understanding how populations respond to environmental change is central to predicting climate vulnerability and guiding conservation interventions (Parmesan and Yohe 2003; Stillman 2003; Umina et al. 2005; CaraDonna et al. 2014). The studies in this Special Issue examine how local adaptive genomic variation is shaped by climate, habitat heterogeneity, and anthropogenic disturbance across a wide range of taxa and ecosystems. Together, they highlight both the power and the limitations of genomic approaches for identifying vulnerable populations and forecasting population responses under rapid environmental change.

Several contributions focus on forest trees, where long generation times and restricted dispersal may limit rapid local adaptation (Gougherty et al. 2021). As foundational components of terrestrial ecosystems, forests play a central role in mitigating climate change and extreme events, yet they are themselves highly vulnerable to environmental change. Consequently, understanding and monitoring forest responses to rapid climate change is a key priority for biodiversity conservation (Feng et al. 2024). Lu et al. (2025) investigate local adaptation and climate vulnerability in the relict conifer *Taiwania cryptomerioides* Hayata, combining genomic analyses with environmental modelling to identify populations likely to become vulnerable under future climates. Their work highlights the importance of balancing local adaptation with assisted gene flow strategies and argues that mixing local and nonlocal seed sources may help maintain adaptive potential in long‐lived tree species. Similarly, Francisco et al. (2025) examine genomic signatures of climate‐driven adaptation and maladaptation in the English yew ( *Taxus baccata* L.), an iconic but increasingly threatened conifer species. By linking genomic variation with climatic gradients, they identify populations potentially at risk under future warming scenarios, particularly in Mediterranean and high‐elevation populations where historical adaptation may become maladaptive.

Adaptive potential in Scots pine ( *Pinus sylvestris* L.) is explored in two complementary studies. Łabiszak and Wachowiak (2025) assess genomic offsets across populations of this keystone and broadly distributed forest species and identify regions likely to experience increased maladaptation under projected climate scenarios in the northeastern European region. Their analysis highlighted that loci associated with climatic variables, in particular annual temperature, are primary drivers of adaptive differentiation. Complementing this landscape genomics perspective, Neuwirthová et al. (2025) investigate drought responses in Scots pine seedlings using high‐throughput phenotyping and SNP‐based analyses. Their study quantifies temporal physiological responses to water stress and demonstrates substantial heritable variation in photosynthesis‐related traits, providing valuable insights for climate‐resilient forestry and breeding programmes in a commercially important forest tree. Buck et al. (2026) extend this approach to the coast live oak ( *Quercus agrifolia* ), a foundation species in California, using whole‐genome data to assess climate adaptedness and guide practical conservation decisions. They identify potentially maladapted populations at the northern and southern range limits, as well as highly adapted but currently unprotected stands along the central coast. Working with The Nature Conservancy, the authors translate these results into recommendations for land acquisition, restoration and within‐preserve seed transfer, illustrating how landscape genomics can support directly actionable climate‐adaptation strategies. Additional studies emphasise how environmental heterogeneity can shape adaptive variation across both large and fine spatial scales. Arenas et al. (2025) demonstrate that both broad‐scale and local‐scale soil chemistry contribute to adaptation in tropical montane forests of sacred fir (*Abies religiosa* (Kunth) Schltdl. & Cham.), a Mesoamerican tree with restricted distribution, emphasising that adaptive responses are often shaped by complex environmental mosaics rather than by single climatic variables alone. Their study not only uncovered an intricate interplay between soil variables and genetic diversity, but also that adaptation to soil chemical factors is polygenic. They conclude by recommending that edaphic variation should be integrated into strategies for the management and conservation of forest trees. Similarly, Lezzi et al. (2026) use landscape genomics to investigate climate adaptation in 28 maize ( *Zea mays* L.) landraces from Italy. Their analyses reveal high genetic diversity, admixture, and candidate adaptive loci associated with environmental variation, highlighting the importance of traditional landraces as reservoirs of stress‐tolerance alleles for breeding climate‐resilient crops. Understanding the factors influencing landrace adaptability is critical for breeders to select alleles linked to environmental adaptation.

Several contributions also explore adaptation and vulnerability in animal systems. Waraniak et al. (2026) investigate genomic responses in the northern leopard frog ( *Rana pipiens* ) across landscapes experiencing rapid climate and land‐use change. Although high levels of gene flow limits strong adaptive structure, agricultural land use emerges as a major driver of genomic variation, while relatively few adaptive variants are linked to these environments. The findings suggest that habitat degradation and land‐use change may currently represent stronger threats to this species than climate change or connectivity loss alone. In marine systems, Nielsen et al. (2025) investigate the combined effects of latitudinal environmental gradients, recent climatic anomalies, and size‐selective harvesting in the intertidal gastropod *Lottia gigantea* G. B. Sowerby I. The study reveals strong genomic differentiation associated with sea surface temperature and pH, particularly between northern and southern range edges, whereas harvesting pressure produced comparatively weak genomic signals. Oosting et al. (2026) similarly demonstrate that extensive population connectivity does not preclude fine‐scale population structure and local adaptation in Australasian snapper ( *Chrysophrys auratus* ). Whole‐genome sequencing of 382 individuals from 13 locations across New Zealand reveals distinct northeastern and southwestern genetic clusters, separated by genomic discontinuities around the Tauroa and Mahia Peninsulas but connected by admixture at their boundaries. The authors identify 38 strongly differentiated genomic regions, including two regions containing growth‐associated candidate genes that exhibit signatures of directional selection in the northeastern cluster and balancing selection in the more environmentally heterogeneous southwestern cluster. A related study by Brauer et al. (2026) also identified environmentally associated population structure in Australian snapper despite the species' prolonged dispersive larval phase. These findings illustrate how connectivity, environmental heterogeneity and contrasting forms of selection can jointly maintain adaptive differentiation in a high‐gene‐flow marine species, highlighting the need to preserve and monitor both standing and adaptive genomic variation under climate change.

### 2.2 Genomic Offsets and Predictive Approaches

A major focus of recent conservation genomics research has been the development of predictive frameworks to forecast maladaptation under future environmental change and guide proactive management interventions. Several contributions in this Special Issue apply genomic offset approaches (Fitzpatrick and Keller 2015), which estimate the mismatch between present‐day adaptive genomic composition and the projected one under future environments (Capblancq et al. 2020, 2023; Rellstab et al. 2021; Lachmuth et al. 2024). Although genomic offset has become a widely used proxy for climate maladaptation, it does not directly estimate future fitness decline and remains sensitive to model choice, marker selection, demographic structure and environmental predictors (Ahrens et al. 2026). These factors therefore need to be carefully evaluated, and genomic offset predictions should be validated against independent measures of fitness before they can be applied operationally (Fitzpatrick et al. 2021; Lachmuth et al. 2023; Archambeau et al. 2026).

In addition to the studies on *Taxus baccata* (Francisco et al. 2025) and *Pinus sylvestris* (Łabiszak and Wachowiak 2025) mentioned above, several other studies in this Special Issue apply genomic offset approaches to better understand future climate vulnerability in forest trees. Fijarczyk et al. (2026) use four long‐term (> 40 years old) common gardens in black spruce ( *Picea mariana* (Mill.) Britton, Sterns & Poggenburg) to directly evaluate whether genomic offset predictions correspond to experimentally measured fitness, addressing a key challenge in validating this approach. Genomic offset estimates were generally robust to model choice, marker type, and training‐set size, although performance varied substantially among common gardens. Predictive accuracy depended also on the climate variables used, their interactions with fitness traits, and the genetic background of populations. Overall, genomic offset showed limited predictive power across the species range, highlighting challenges associated with spatially variable adaptation in genetically structured species. In another study, Morando‐Milà et al. (2026) investigate adaptive variation and future climate vulnerability in European beech ( *Fagus sylvatica* L.) populations across the Mediterranean range margin. The study identifies hundreds of climate‐associated SNPs linked to drought and temperature gradients, and predicts strong maladaptation hotspots under severe warming scenarios. Importantly, the authors introduce the Required Evolutionary Rate (REvoRate), a novel metric estimating the minimum rate of genetic change needed for populations to track future climates, under the assumption that current observations reflect expectations for local adaptation equilibrium. Similarly, Sekely et al. (2026) examine future maladaptation in the Patagonian tree *Nothofagus pumilio* (Poepp. & Endl.) Krasser using paired‐site genomic analyses across fine‐scale environmental gradients. Their results reveal highly heterogeneous patterns of genomic offset that do not follow simple elevational or latitudinal trends, emphasising the importance of local environmental variation for conservation planning and assisted gene flow strategies. A complementary study on *Populus tremuloides* Michx. by Zacharias et al. (2026) further evaluates future maladaptation risk in this widespread keystone forest species, reinforcing the growing application of predictive genomic approaches in forestry and restoration contexts. In this study, genomic offsets were greatest in Mexican and southwestern U.S. populations, pointing to higher projected vulnerability, whereas northern populations showed lower offsets, consistent with a potential northward shift in range. A tentative positive association between polyploidy and genomic offset further points to a possible adaptive or buffering role for polyploidy, a pattern that warrants further investigation.

Several contributions also explore how the genomic offset framework can inform applied management in breeding programmes. Beck, Kess, et al. (2025) investigate the genomic basis of migration timing in Atlantic salmon ( *Salmo salar* L.) and identify a polygenic architecture involving candidate genes associated with adaptation for this trait. By integrating these findings with genomic offset analyses, the study predicts which populations and migration phenotypes are most vulnerable under future climate change, particularly late‐run and multiple‐peak migration systems. Jacquemart et al. (2025) study the genomic resources for kokanee salmon ( *Oncorhynchus nerka* (Walbaum)) to develop climate‐informed stocking strategies across British Columbia. Using relatively small panels of climate‐associated markers, the study identifies suitable donor and recipient populations and provides applied tools for freshwater fisheries management under climate change. Applying some of these concepts to genomics‐informed selective breeding programmes, Beck, May, et al. (2025) propose an ‘adaptive breeding framework’ that integrates genomic offset predictions into agricultural and aquaculture breeding programmes. The study outlines how approaches developed in evolutionary ecology can help identify resilient genotypes and reduce future climate‐associated production risks, while also discussing practical limitations and implementation challenges.

### 2.3 Mechanisms of Adaptation and Functional Genomics

Understanding adaptive potential requires moving beyond neutral patterns of genomic diversity toward identifying the functional variants, regulatory pathways, and molecular mechanisms that underpin fitness‐related traits (Wu and Lin 2006). Several studies in this Special Issue investigate the genetic architecture of adaptation through integrative approaches combining natural history and ecology with genomics, transcriptomics, metabolomics, epigenetics and high‐throughput phenotyping. Together, these contributions demonstrate that adaptive responses to environmental change are often mediated by complex interactions among standing genetic variation, gene regulation, phenotypic plasticity and epigenetic processes.

Several contributions focus on plant responses to climatic stress. Gil‐Muñoz et al. (2025) investigate how long‐term breeding for growth in Scots pine ( *Pinus sylvestris* L.) influences drought tolerance using metabolomics, hormone profiling and image‐based phenotyping. Their results show that bred populations can exhibit increased drought resistance but often contain reduced genetic variation relative to natural forests, highlighting potential trade‐offs between productivity and adaptive capacity. Tiret et al. (2025) examine germination responses to high temperatures across *Brassica* species, including landraces and wild relatives, and reveal substantial previously unexplored variation associated with species identity and geographic origin. The study identifies germination as an important but underutilised trait for developing crop resilience under future warming scenarios. Furthermore, Collins et al. (2025) investigate genomic and physiological variation in the tropical wetland tree *Pterocarpus officinalis* Jacq. and identify substantial differences among seed sources in foliar and water‐use efficiency (WUE) traits despite low overall genetic differentiation. Trait variation is associated with climatic variables such as precipitation and elevation, and genome‐wide association analyses identify candidate loci linked to stress response, metabolism and epigenetic regulation. Together, these studies demonstrate how integrating functional traits, genomics and environmental data can help identify populations and traits relevant to climate‐adaptive restoration and breeding strategies.

Other studies highlight the importance of regulatory and epigenetic mechanisms in rapid responses to environmental changes, processes that are increasingly recognised to be important in evolutionary adaptation (Venney et al. 2023). Yan et al. (2025) use a multi‐omics approach in the invasive tunicate *Ciona robusta* Hoshino & Tokioka to demonstrate complementary roles of DNA methylation and microRNAs in regulating gene expression under repeated salinity stress. Their findings suggest that epigenetic and post‐transcriptional regulatory mechanisms can jointly facilitate rapid phenotypic plasticity during biological invasions. Moreover, Attfield et al. (2026) synthesise evidence showing how captive rearing environments induce epigenetic changes in salmonids that affect behaviour, morphology, coloration and life‐history traits. The authors further discuss how epigenetic biomarkers and environmentally informed hatchery practices could improve the success of supplementation and conservation programmes under changing climatic conditions.

Additional contributions in bacteria and algae explore how adaptive potential emerges through interactions among environmental stress, genomic diversity and ecological dynamics. Kaur et al. (2025) investigate pathogen evolution in agroecosystems using strain‐resolved metagenomics of *Xanthomonas* infecting pepper under elevated ozone stress. Their results show that climatic stress can maintain pathogen diversity, promote strain turnover, and alter the balance between standing variation and de novo mutation, thereby influencing the durability of host resistance. Echenique‐Subiabre et al. (2025) examine adaptive responses in *Nannochloropsis* following long‐term outdoor cultivation across contrasting climatic conditions. In contrast to expectations of strong local adaptation, the study finds limited site‐specific genomic divergence, with seasonal effects and broad phenotypic plasticity appearing to buffer populations against directional selection. These findings emphasise that persistence under environmental change may depend not only on local adaptation but also on plasticity and fluctuating selection regimes.

Finally, Tan et al. (2026) evaluate genomic predictors of inbreeding depression in the recovering New Zealand hihi/stitchbird ( *Notiomystis cincta* (Du Bus)). By linking genomic metrics such as runs of homozygosity, realised load, and genome‐wide homozygosity to lifetime reproductive success, the study demonstrates that genome‐wide homozygosity remains the strongest predictor of fitness differences, likely reflecting the cumulative effects of many small‐effect loci. The work highlights both the potential and the complexity of applying genomic metrics to the conservative management of small and recovering populations.

### 2.4 Population Genomics, Structure and Demography

Understanding how selection, gene flow, and demographic history interact to shape genomic variation remains a central challenge in evolutionary ecology and conservation genomics (Tiffin and Ross‐Ibarra 2014; Schiebelhut et al. 2024). This is particularly important because the genomic variation available to natural and artificial selection provides the raw material for evolutionary change and adaptation. Effective population size (N_e_) links demographic history to this variation: it reflects the strength of genetic drift and helps track how genetic diversity is maintained or lost over time. When combined with genomic data, N_e_ estimates can support conservation monitoring and management by tracking changes in genetic diversity and population history (Allendorf 2025; Mergeay et al. 2026). The studies in this section investigate how contemporary and historical processes structure populations across environmental gradients, influence adaptive potential, and shape vulnerability under ongoing environmental change (Capblancq et al. 2020). Together, they demonstrate that genomic patterns often reflect the combined effects of local adaptation, connectivity, demographic bottlenecks, and historical range dynamics, making integrative population genomic approaches essential for effective conservation and management.

Several contributions focus on the conservation of fragmented or threatened populations. Balkwill et al. (2025) investigate adaptive potential in the critically endangered New Zealand tree *Syzygium maire* (A.Cunn.) Sykes & Garn.‐Jones. The study identifies substantial genomic variation across environmental gradients and proposes five conservation management units for the species. Importantly, the authors highlight that maintaining or restoring connectivity among fragmented populations will be critical for long‐term persistence, while also emphasising the risks associated with incomplete knowledge of geographic adaptive variation in threatened tree species. An animal study further demonstrates how demographic history and connectivity shape contemporary genomic structure. Liu et al. (2026) examine population structure in the marine fish *Evynnis cardinalis* (Lacépède) along the Chinese coast using whole‐genome sequencing. Despite broad geographic sampling, the species exhibited striking genomic homogeneity, suggesting high connectivity and extensive gene flow across coastal populations. These findings highlight how strong dispersal and demographic connectivity can maintain genomic cohesion even across environmentally heterogeneous marine systems, and emphasise the application of high‐density and high‐resolution markers in determining fish population structure.

Other studies further investigate how demographic decline and historical processes leave detectable genomic signatures in vulnerable populations. Schnelle et al. (2026) compare historic and contemporary genomes of endangered gull‐billed tern ( *Gelochelidon nilotica* (Gmelin, JF)) populations using both museum specimens and modern samples. While overall nucleotide diversity remained relatively stable, contemporary populations exhibit elevated inbreeding and reduced heterozygosity compared to historical populations, reflecting the genomic consequences of recent demographic decline and fragmentation. Effective population size analyses further reveal multiple historical bottlenecks, emphasising the importance of long‐term monitoring to prevent future inbreeding depression. Fenton et al. (2026) investigate population genomic structure in Arctic charr ( *Salvelinus alpinus* L.) across Britain and Ireland, revealing extensive population subdivision with most lake populations representing distinct genetic clusters. At broader scales, however, genomic structure reflects historical drainage patterns and postglacial colonisation history. The study also identifies southern and inland populations as particularly vulnerable under future climate change and proposes Evolutionarily Significant Units (ESUs) to prioritise conservation of this highly diverse species complex. Finally, Chen et al. (2025) provide a different perspective by examining selective filtering during biological invasions in the ascidian *Ciona robusta* Hoshino & Tokioka. Using whole‐genome resequencing, the authors demonstrate that high‐salinity stress during maritime transport acts as a strong selective filter, driving genotype‐dependent mortality and allele frequency shifts in osmoregulatory genes. The study challenges the assumption that maritime transport represents a neutral demographic bottleneck and instead shows that selection can begin acting at very early invasion stages.

### 2.5 Applied Conservation, Management and Restoration

The final section of this Special Issue highlights how genomic and evolutionary approaches are increasingly being translated into practical conservation, restoration and management applications (Allendorf et al. 2010). Across terrestrial, freshwater and marine systems, these studies demonstrate how genomic data can guide decisions about extinction‐risk assessment, assisted gene flow, restoration design, seed sourcing, population monitoring and climate adaptation planning. Together, they illustrate a broader shift from purely descriptive conservation genetics toward predictive and intervention‐oriented frameworks that explicitly integrate evolutionary processes into management.

A recurring theme across several contributions is the challenge of managing maladaptation under rapid environmental change. One increasingly discussed intervention is assisted gene flow (AGF), which involves the deliberate movement of individuals or propagules within a species' historical range to enhance population resilience (Aitken and Whitlock 2013; Aitken and Bemmels 2016). AGF may increase genetic diversity and reduce inbreeding through genetic rescue while also introducing pre‐adapted alleles that facilitate evolutionary rescue under novel environmental conditions. However, despite growing interest, major uncertainties remain regarding implementation, including the optimal frequency and magnitude of introductions, long‐term demographic outcomes, and the balance between adaptive benefits and risks such as genetic swamping or outbreeding depression. These concerns are especially important because introduced genotypes could potentially dilute locally adapted variation or disrupt co‐adapted gene complexes. At the same time, several studies in this issue illustrate that careful genomic characterisation can substantially improve the design of such interventions and reduce risks.

In plants, multiple studies demonstrate how genomic and phenotypic information can inform climate‐adaptive restoration and afforestation strategies. Kilkenny et al. (2026) model climate‐associated trait variation and plasticity in the restoration grass *Elymus elymoides* (Raf.) Swezey, identifying substantial differences among populations in growth, morphology, phenology and survival responses across environmental conditions. Importantly, the study identifies 13 climate‐informed seed zones and introduces a prioritisation framework that balances adaptive matching with practical restoration constraints, providing a scalable template for seed sourcing under future climates. Similarly, Friis et al. (2025) examine the genetic consequences of tree planting versus natural colonisation in *Quercus robur* L. (pedunculate oak) and *Betula pendula* Roth (silver birch) within UK afforestation programmes. While planted stands retained comparable overall genetic diversity, they exhibited elevated co‐ancestry. In addition, commonly used registered seed sources captured only part of the species' full bioclimatic variation. These findings highlight how restoration strategies that appear successful demographically may nevertheless constrain long‐term adaptive potential under climate change. Szamosvári et al. (2026) further demonstrate that genetic ancestry can strongly influence ecologically important traits in hybridising forest trees. Using common‐garden experiments involving *Populus alba* L., *Populus tremula* L. and their natural hybrid *Populus × canescens* (Aiton) Sm., they show that ancestry affects juvenile survival, vegetative growth, clonal rooting success and leaf pubescence. Hybrids with greater *P. alba* ancestry generally exhibited enhanced early growth and clonality, whereas recombinant hybrids and backcrosses to *P. tremula* experienced higher juvenile mortality. These results illustrate both the opportunities and risks associated with using hybrids in restoration, afforestation and forest breeding, and highlight the importance of considering ancestry‐dependent fitness rather than assuming that all hybrid genotypes are functionally equivalent. Additional insights into restoration under future climates are provided by Collins et al. (2025), who investigate the genomic and physiological basis of structural and foliar trait variation in the tropical tree *Pterocarpus officinalis* . Their work links functional trait variation with adaptive genomic differences relevant to drought resilience, directly informing restoration planning under projected drying conditions. Likewise, Provazník et al. (2025) demonstrate how hyperspectral phenotyping can detect adaptive genetic variation in Scots pine ( *Pinus sylvestris* L.) across sites and seasons. By showing that remotely sensed spectral traits reliably capture underlying genetic differences, the study provides an important proof‐of‐concept for scalable forest monitoring and climate‐adaptive breeding approaches that extend beyond controlled experimental systems.

Studies in marine species further demonstrate how genomic data can directly inform restoration and management decisions in highly connected coastal ecosystems. Jahnke et al. (2025) show that adaptive and functionally important variation in eelgrass ( *Zostera marina* L.) can occur even among meadows separated by only a few kilometres. By integrating population genomics, environmental heterogeneity, and experimental performance data, the study challenges the assumption that nearby donor populations are interchangeable and instead supports restoration strategies that explicitly consider local adaptation and resilience to future stressors. Similarly, Jenkins et al. (2025) investigate clonal diversity, population structure and potential adaptation to environmental gradients in the habitat‐forming coralline algae *Phymatolithon calcareum* (Pallas) W. H. Adey & D. L. McKibbin and *Lithothamnion corallioides* (P. Crouan & H. Crouan), which create ecologically important maerl beds in the Northeast Atlantic. Their analyses reveal strong spatial structure in clonal diversity and dispersal limitation, identify populations potentially vulnerable under future climates, and highlight candidate donor populations for future restoration initiatives.

Coral reef systems feature prominently in this section, reflecting the urgent need to identify climate‐resilient populations under increasing marine heatwave pressure. Machine‐learning‐based population genomics was used to investigate environmental drivers of genomic variation in two coral species across the Florida Keys Reef Tract (Black et al. 2025). While thermal stress contributed to genomic differentiation, the study surprisingly found that depth and water chemistry, including nutrient concentrations and salinity, explained substantially more variation than temperature alone. These findings caution against overly simplistic restoration strategies focused exclusively on thermal tolerance and instead emphasise the importance of considering multidimensional environmental variation when selecting source populations for assisted gene flow and transplantation. Complementing this work, Guillaume et al. (2026) investigate coral population structure across the Western Indian Ocean and show that ocean circulation patterns strongly shape connectivity and adaptive divergence in *Acropora muricata* L. and *Pocillopora damicornis* L. Using genotype–environment association (GEA) analyses, the authors identify hundreds of loci associated with temperature stress responses, while also demonstrating that isolation‐by‐resistance linked to oceanographic barriers better explains genomic structure than geographic distance alone. Together, these studies reinforce the importance of integrating both environmental heterogeneity and dispersal processes into marine conservation planning. In freshwater systems, a study by Barratt et al. (2025) combines genomic offset predictions, admixture analyses, connectivity inference, and occupancy‐based species distribution models in an East African reed frog species complex (*Hyperolius* spp.). Their integrative framework identifies genomically distinct management units while disentangling risks associated with future maladaptation versus habitat fragmentation. Importantly, the study provides an open‐source and scalable analytical pipeline that can guide conservation prioritisation and intervention strategies across complex landscapes and taxonomic groups.

Finally, this section concludes with a broader conceptual and thought‐provoking perspective by Mariette and Gerber (2025), who critically reflect on the environmental footprint, assumptions, and policy implications of increasingly data‐intensive population genomic research. Their perspective challenges the field to consider not only what genomics technologies can achieve, but also the material and societal costs associated with large‐scale sequencing efforts and conservation genomics infrastructure. In doing so, the article raises important questions about sustainability, scalability, and the relationship between knowledge generation and meaningful conservation action.

## 3 Outstanding Questions and What's Next

This Special Issue highlights the remarkable progress that has been made in understanding the adaptive potential of populations in the face of rapid environmental change, but it also makes clear that major conceptual, empirical, and practical challenges remain (Box 1). Across systems and taxa, contributions consistently emphasised that climate adaptation is not solely a biological problem related to genetics, but an integrated ecological, evolutionary, social and governance challenge.

One of the most pressing outstanding questions concerns how studies on the genomics of adaptation can be translated into practical conservation and management action under conditions of accelerating climate change and high uncertainty. Advances in evolutionary genomics, large‐scale sequencing, environmental association analyses, predictive modelling, and experimental approaches are rapidly improving our ability to identify adaptive variation (Ekblom and Galindo 2011; Ellegren 2014; Tagu et al. 2014). However, substantial uncertainty remains regarding how these insights should guide intervention strategies in real‐world systems. Several contributions highlight the need to move beyond documenting genomic patterns toward understanding the mechanistic and fitness consequences of adaptive variation across generations and environmental contexts. It is important to distinguish adaptive potential, which is related to standing genetic variation in adaptive traits (and to a lesser extent, mutation) and any potential mismatch between current and future environmental conditions and spatial allele frequency patterns. It remains much more common in the literature to focus research on the latter, but quantifying the former is also important.

A recurring theme across papers is the growing tension between traditional preservation‐focused conservation philosophies and more interventionist approaches aimed at facilitating adaptation under climate change. Resilience‐oriented breeding, AGF, genomic‐informed restoration, and climate‐adapted translocations are increasingly discussed as necessary tools to maintain resilience in rapidly changing environments. Yet their implementation continues to face sociological, philosophical, and governance barriers that may ultimately prove more difficult to overcome than technical limitations themselves. Debates over ‘how local is native?’ remain central, particularly where the introduction of non‐local genotypes challenges views of ecological authenticity and acceptable restoration practice (Breithaupt et al. 2026). There is also a need to consider the financial cost/benefit of deploying genomic tools relative to other conservation interventions (e.g., habitat conservation), a problem which requires interdisciplinary collaboration across many other fields.

Importantly, the Special Issue also underscores that inaction itself carries substantial risks. Species are already experiencing range contractions, local extirpations, altered phenologies, and reduced fitness under contemporary climate conditions, and this collection highlights how genomic approaches can uncover patterns as well as the processes across diverse species. While concerns surrounding evolutionary interventions are frequently raised, empirical evidence demonstrating widespread negative outcomes remains comparatively limited relative to the existing and growing evidence of climate‐driven biodiversity loss. This raises an important future challenge for the field: developing evidence‐based frameworks that allow managers to evaluate the relative risks of intervention versus non‐intervention under uncertain future climates and long‐term evolutionary trajectories.

Another major unresolved issue is how success should be defined and measured in applied evolutionary management. Relevant metrics are inherently dependent on the species, ecosystem, the services they provide and stakeholder priorities (Parmesan et al. 2022) and often involve trade‐offs across levels of biological organisation. In some systems, success may be reflected in improved reproductive output, population growth, or individual resilience to environmental stressors. In others, maintaining ecosystem function, preserving rare endemic diversity, or sustaining cultural and economic values may take precedence. Conservation outcomes therefore cannot be determined by biological data alone; they require transparent dialogue among scientists, managers, Indigenous communities, policymakers and broader society. A further challenge is balancing the need for consistent, comparable metrics with the flexibility to tailor assessment frameworks to the factors most relevant to each study system.

Several contributions further emphasised the need for longer‐term and more integrative studies capable of disentangling the relative contributions of standing genetic variation, plasticity, epigenetic responses, demographic history, and evolutionary adaptation to species and population persistence. In particular, combining field‐based genomic studies with controlled experiments (e.g., common garden or reciprocal transplants) emerged as a powerful but still underutilised approach for linking genomic signatures to functional fitness consequences under realistic environmental conditions. Future work integrating genomics with physiology, ecology, epigenomics, and predictive modelling will be critical for improving forecasts of species resilience and identifying populations most vulnerable to future environmental change under realistic land‐use and management scenarios. Moreover, most studies in the Special Issue focus on a single target organism, despite species being embedded in complex ecosystems where biotic interactions, including competition, complementarity, and facilitation, can strongly influence resilience. Multi‐species and, ultimately, whole‐ecosystem approaches are therefore urgently needed to better understand how biodiversity and species interactions shape responses to environmental change.

Finally, this Special Issue highlights a broader conceptual transition currently occurring within conservation and management. Rather than viewing ecosystems as static entities to be preserved in historical states, something which will likely not be possible anyway, there is an increasing recognition that adaptive stewardship approaches may be necessary in an era of unprecedented environmental change. Such approaches acknowledge that maintaining biodiversity and ecosystem function under future climates may require dynamic, forward‐looking strategies that explicitly incorporate evolutionary processes and adaptive potential into management frameworks.

Together, the papers assembled in this Special Issue demonstrate that evolutionary genomics is rapidly moving from a primarily descriptive discipline toward one increasingly capable of informing actionable conservation and management decisions. Realising this potential will require continued integration across disciplines, stronger connections between empirical data and applied decision‐making, and sustained engagement with the ethical, social, and governance dimensions of climate‐adaptation interventions.

BOX 1 **Outstanding Questions.**

**Table:** 

How accurately can genomic predictions forecast long‐term fitness and resilience under future climates?
2 Which traits underlying historical local adaptation will remain important under rapidly changing environmental conditions?
3 To what extent can phenotypic plasticity buffer populations against climate change relative to genetic adaptation, and what are the relevant timescales for evaluating whether plasticity is adaptive versus maladaptive?
4 How can genotype–environment–fitness relationships be quantified at scales relevant for real‐world conservation and restoration?
5 How can we move from predicting the vulnerability of individual species to predicting the resilience and adaptive capacity of interacting ecological communities?
6 What are the long‐term evolutionary and ecological consequences of assisted gene flow and climate‐adapted translocations?
7 How should managers balance the risks of intervention against the risks of non‐intervention under accelerating environmental change?
8 Which populations, including trailing‐edge and climatically extreme populations, contain adaptive variation most critical for future resilience?
9 How can genomics be more effectively integrated into operational restoration, forestry, fisheries and conservation management frameworks?
10 How can conservation strategies better incorporate ecological complexity, social values, Indigenous knowledge, and governance challenges alongside genomic data?

## 4 Conclusions

This Special Issue demonstrates that adaptive potential is shaped by complex interactions among genomic variation, climate, demography, connectivity, plasticity and ecological context, while many populations already exhibit signals of maladaptation to future climates. A major challenge now is to translate genomic insights into real‐world management and applications. Despite rapid advances in sequencing and downstream spatial, temporal and environmental association analytical and predictive frameworks, empirical data and expectations for the relationships directly linking genotypes, environments and fitness remain limited for most species and contexts. As a result, conservation and management decisions surrounding application of genetic indicators, assisted gene flow, restoration and climate adaptation are still often made under substantial uncertainty. However, inaction also comes at a cost. The studies carefully explore how evidence‐based direct approaches and interventions can help halt biodiversity loss and range contractions while sustaining food production and a wide range of ecosystem services under ongoing environmental change.

The contributions further highlight that adaptation cannot be reduced to simple climate matching. Future resilience will depend on maintaining ecological interactions, phenological timing, ecosystem function and composition, and adaptive flexibility under changing environments. Integrating genomics with physiology, phenotyping, experiments and environmental modelling will therefore be critical for developing robust predictive and intervention‐based conservation strategies. Together, these studies provide a strong call for rigorous, mechanistic and applied science that bridges evolutionary genomics with practical conservation and restoration in the Anthropocene.

## Conflicts of Interest

Some of the authors of this article are Editorial Board members of Evolutionary Applications. To minimise bias, they were excluded from all editorial decision‐making related to the acceptance of this article for publication.

## Data Availability

Data sharing not applicable to this article as no datasets were generated or analysed during the current study.
